# Interpretable machine learning for identifying cost drivers of inpatient traditional Chinese medicine services in a tertiary hospital in China

**DOI:** 10.3389/fpubh.2026.1937712

**Published:** 2026-10-21

**Authors:** Xi Li, Shouyan Chen, Mei Gong, Changyang Chen, Jia Chen, Jiangping Zhang

**Affiliations:** 1 School of Public Health, Key Laboratory of Environmental Pollution Monitoring and Disease Control, Ministry of Education, Guizhou Medical University, Guiyang, Guizhou, China; 2 Guiyang Public Health Treatment Center, Guiyang, Guizhou, China

**Keywords:** cost prediction, machine learning, patient cost, public health, traditional Chinese medicine

## Abstract

**Background:**

Inpatient traditional Chinese medicine (TCM) costs are an important component of hospital expenditure in China, yet their cost structure and drivers remain poorly characterized. This study aimed to identify the drivers of total TCM cost among inpatients in the TCM department of a tertiary hospital using interpretable machine learning.

**Methods:**

This retrospective study used 1,532 de-identified inpatient records (2023–2025) from the TCM department of a tertiary hospital in Guiyang, China. Because the model includes cost-component variables (the total cost of TCM treatment items and the total cost of Chinese herbal medicines) as predictors, it is framed explicitly as a cost-structure explanatory model rather than a prospective prediction model, and its explanatory power partly reflects these cost components. Because the outcome was right-skewed, total TCM cost was log(1 + x)-transformed. Seven algorithms (linear, ridge, lasso, elastic net, decision tree, random forest and XGBoost regression) were compared using repeated 5-fold cross-validation (5 repeats) and an independent 8:2 hold-out test set. Model performance was assessed by RMSE, MAE and *R^2^*, and permutation importance and Shapley additive explanations (SHAP) quantified variable contributions.

**Results:**

The cohort had a median age of 61 years and 61.4% female patients; the median total TCM cost was 3,446.72 yuan (IQR 1,616.00–5,324.05). The random forest achieved the best performance (test *R^2^* = 0.820 on the log scale; back-transformed RMSE = 1,408.6 yuan, *R^2^* = 0.746), outperforming XGBoost and all regularized linear models. Permutation importance and SHAP consistently ranked the total cost of TCM treatment items, length of hospital stay and the total cost of Chinese herbal medicines as the three leading predictors (approximately 81% of the total SHAP importance), whereas demographic and clinical factors contributed little.

**Conclusion:**

The total cost of TCM treatment items is the dominant driver of total TCM cost, followed by length of hospital stay and the cost of Chinese herbal medicines. These findings support refined cost accounting and standardized management of TCM treatment items. The reported contributions reflect associations within the model rather than causal effects; given the single-center design and internal validation only, multicenter external validation and admission-stage models are required before any clinical or administrative use.

## 1 Introduction

The continuous increase in medical expenditure has become an important challenge for health systems worldwide. In China, this issue is particularly relevant in the context of population aging, the growing burden of chronic diseases, and ongoing reforms in medical insurance payment systems (1). Under the Healthy China 2030 strategy and the implementation of diagnosis-related group (DRG) and diagnosis-intervention packet (DIP) payment reforms, hospitals are increasingly required to improve cost control, optimize resource allocation, and maintain service quality (2, 3).

Traditional Chinese medicine (TCM) services are widely used in China and are increasingly integrated into chronic disease management, rehabilitation, and inpatient supportive care. In hospital settings, the cost structure of TCM departments may differ from that of departments dominated by conventional Western medical services. For inpatients receiving TCM care, the costs of TCM-specific diagnostic and therapeutic services, Chinese herbal medicines, Western medicines, examinations, nursing, and other inpatient services may be incurred. Understanding the relative contribution of these cost components is important for refined cost accounting, payment policy design, and evidence-informed management of TCM services (4–6).

Previous studies of hospitalization costs have mainly focused on specific diseases, surgical procedures, or Western medicine-oriented inpatient care. However, evidence on cost structure and cost drivers among patients admitted to TCM departments of general hospitals is limited (7, 8). In particular, few studies have explored the relative contribution of TCM treatment item costs, Chinese herbal medicine costs, Western medicine costs and patient-level characteristics within the same analytical framework (9, 10).

Machine learning methods offer flexible tools to model complex, nonlinear relationships in heterogeneous clinical and administrative data. Random forest regression in particular can accommodate nonlinear associations and interactions in the data without strong parametric assumptions. In addition to prediction, permutation-based variable importance can help identify which variables contribute most to model performance. However, when cost-component variables are used to explain total cost, the model should be interpreted carefully because high explanatory performance may partly reflect the arithmetic relationship between total cost and its components rather than true prospective prediction (11–13).

Therefore, this study aimed to identify the drivers of total TCM cost among inpatients admitted to the TCM department of a tertiary hospital in Guiyang, China, using interpretable machine learning. To address the right-skewed distribution of cost data, the outcome was log(1 + x)-transformed. Repeated 5-fold cross-validation (5 repeats) with hyperparameter tuning was used for model development, and an independent 8:2 hold-out test set was retained for out-of-sample verification. Model predictive efficacy was quantified using RMSE, *R^2^* and MAE, with overfitting screened by comparing training, cross-validation and test-set performance. We further quantified the relative predictive importance of cost components and patient sociodemographic/clinical covariates via SHAP interpretation and variable importance analyses, to generate evidence supporting refined cost accounting and targeted cost management for inpatient TCM services.

## 2 Materials and methods

### 2.1 Study design and data source

This retrospective hospital-based observational study was conducted using inpatient medical record data from the Department of TCM of a tertiary hospital in Guiyang, China. The study period covered admissions between 2023 and 2025. Data were extracted from the hospital information system and de-identified using unique study codes before analysis. The unit of analysis was the hospitalization record. No repeated hospitalization records exist for the same patient, and each admission record is independent.

### 2.2 Inclusion and exclusion criteria

Hospitalization records were eligible if patients were admitted to the TCM department during the study period, had complete hospitalization cost information, and had available demographic, clinical, and payment-related variables required for modeling. Records were excluded if key clinical or cost variables were missing, if the proportion of out-of-pocket expenses was outside the logical range of 0 to 1, or if total TCM cost values were judged to be extreme or clinically implausible after verification.

A total of 1,583 hospitalization records were initially identified. After excluding records with missing key variables, implausible cost values, out-of-pocket payment proportions outside the range of 0 to 1, and extreme values after statistical and clinical verification, 1,532 records were included in the final analysis. The detailed inclusion and exclusion process is shown in Supplementary Figure S1.

### 2.3 Variables and outcomes

The outcome was the total TCM cost per hospitalization, measured in Chinese yuan, defined as the sum of all TCM-related charges (the total cost of TCM treatment items, the total cost of Chinese herbal medicines, and other TCM-specific service fees). Candidate predictors included demographic characteristics, payment-related variables, hospitalization characteristics, and TCM cost-component variables. Categorical variables included ethnicity, payment method, sex, marital status, occupation, and critical illness status. Continuous variables included age, number of hospitalizations, length of hospital stay, proportion of out-of-pocket expenses, total cost of TCM treatment items, and total cost of Chinese herbal medicines.

Because the total cost of TCM treatment items and the total cost of Chinese herbal medicines are components of total TCM cost, this model is interpreted as a cost-structure explanatory model rather than a prospective prediction model, and its explanatory performance partly reflects the arithmetic relationship between total TCM cost and these components. For prospective administrative use, a separate admission-stage prediction model should be developed using only variables available at or shortly after admission, such as age, sex, payment method, admission diagnosis, critical illness status, comorbidities, and planned treatment category.

### 2.4 Statistical analysis

All statistical analyses were performed using R software (version 4.4.1). Continuous variables were first examined for distributional characteristics. Normally distributed variables were summarized as mean ± standard deviation, whereas skewed variables were summarized as median and interquartile range. Categorical variables were presented as frequencies and percentages. Because medical cost data are typically right-skewed, nonparametric tests were prioritized for group comparisons. The Mann–Whitney U test or Kruskal–Wallis test was used for continuous outcomes across groups, and the chi-square test or Fisher’s exact test was used for categorical variables, as appropriate. Spearman correlation coefficients were used to assess monotonic associations between continuous variables and total TCM cost.

Construction and validation of prediction models were conducted. Missing values were imputed before analysis. Because medical costs are typically right-skewed, the outcome (total TCM cost) was transformed as log(1 + cost), and the two skewed cost-component predictors were transformed in the same way. The dataset was randomly split into training and test sets at an 8:2 ratio using a fixed random seed. We built seven models including Elastic Net, Lasso, Linear Regression, Ridge Regression, XGBoost, Random Forest and Decision Tree. All models underwent hyperparameter tuning via repeated 5-fold cross-validation (5 repeats) on the training set. Model performance was evaluated using RMSE, MAE and *R^2^* on the log-transformed scale, and RMSE and MAE were additionally reported in yuan after back-transformation; the optimal model was determined accordingly. Overfitting was assessed by comparing training, cross-validation and test-set performance. The final model was validated on the independent test set. We interpreted the model using variable importance plots, predicted-actual scatter plots, residual analysis and SHAP analyses (feature importance, swarm and dependence plots). SHAP results are interpreted as predictive associations within the model rather than causal effects. The significance level was set at *α* = 0.05.

## 3 Results

### 3.1 Study population and cost distribution

As shown in Table 1, a total of 1,532 hospitalization records were included in the final analysis. The median total TCM cost was 3,446.72 yuan (IQR 1,616.00–5,324.05). Most patients were Han Chinese (93.1%), and women accounted for 61.4% of the study population. The most common payment categories were Basic Medical Insurance for Urban Employees (40.0%), other payment methods (37.5%), and fully out-of-pocket payment (22.4%). Most patients were classified as general rather than critically ill patients.

**Table 1:** Baseline characteristics and correlations with total TCM costs of included patients.

Characteristic	Category	*n* (%)	Total TCM cost, median (IQR), yuan	Statistic	Median (P25, P75)	Spearman correlation	*p* value
Sex	Male	591 (38.6)	3193.08 (1444.43, 4935.53)	*χ^2^* = 5.804			0.016
	Female	941 (61.4)	3604.80 (1712.02, 5520.60)				
Age, years					61 (47, 71)	*ρ* = 0.407	<0.001
Marital status	Married	1,252 (81.7)	3620.15 (1933.34, 5448.32)	*χ^2^* = 134.275			<0.001
	Unmarried	154 (10.1)	357.15 (47.62, 2654.36)				
	Divorced or widowed	81 (5.3)	3756.78 (1867.18, 5685.76)				
	Others	45 (2.9)	4722.98 (1552.50, 5897.48)				
Ethnicity	Han nationality	1,426 (93.1)	3526.15 (1704.55, 5359.67)	*χ^2^* = 14.610			<0.001
	Ethnic minority	106 (6.9)	2101.93 (327.56, 4554.24)				
Occupation	Employed	163 (10.6)	2567.63 (1097.83, 4525.17)	*χ^2^* = 75.414			<0.001
	Flexible employment	126 (8.2)	2188.14 (282.60, 4200.04)				
	Unemployed / Non-employed	359 (23.4)	3103.84 (646.90, 4938.64)				
	Others	884 (57.7)	3797.40 (1973.33, 5832.40)				
Payment method	Urban employee basic medical insurance	613 (40.0)	3829.00 (2130.40, 5970.59)	*χ^2^* = 180.425			<0.001
	New Rural Cooperative Medical Scheme	1 (0.1)	3923.68 (3923.68, 3923.68)				
	Fully out-of-pocket	343 (22.4)	806.40 (71.43, 3794.57)				
	Others	575 (37.5)	3810.62 (2221.32, 5320.09)				
Critical illness status	Yes	67 (4.4)	3830.01 (2745.49, 5945.59)	*χ^2^* = 5.177			0.023
	No	1,465 (95.6)	3413.57 (1557.76, 5282.89)				
Number of hospitalizations					1 (1, 2)	*ρ* = 0.216	<0.001
Length of hospital stay, days					13 (8, 16)	*ρ* = 0.566	<0.001
Proportion of out-of-pocket expenses					0.037 (0.014, 0.109)	*ρ* = −0.245	<0.001
Total cost of TCM treatment items, yuan					3056.35 (1024.28, 4805.69)	*ρ* = 0.848	<0.001
Total cost of Chinese herbal medicines, yuan					10.84 (1.15, 22.89)	*ρ* = 0.633	<0.001

*χ*^2^, Kruskal–Wallis test statistic comparing total TCM cost across categories; ρ, Spearman correlation coefficient between the continuous variable and total TCM cost. IQR, interquartile range; P25/P75, 25th/75th percentiles.

Total TCM cost differed significantly by sex (*p* = 0.016), marital status, ethnicity, occupation and payment method (all *p* < 0.001), and by critical illness status (*p* = 0.023). Among continuous variables, age, number of hospitalizations, length of hospital stay, the total cost of TCM treatment items and the total cost of Chinese herbal medicines were positively associated with total TCM cost, whereas the proportion of out-of-pocket expenses showed a weak negative association with total TCM cost.

### 3.2 Random forest model and SHAP analysis for total TCM cost

We tested seven algorithms to develop the optimal explanatory model for total TCM cost, namely Random Forest, XGBoost, Elastic Net, Lasso Regression, Linear Regression, Ridge Regression, and Decision Tree. As shown in Table 2, the optimized Random Forest model exhibited the best performance in the internal test set, with the lowest RMSE (0.666) and the highest *R^2^* (0.820) and MAE (0.353) on the log-transformed scale, followed closely by XGBoost (RMSE = 0.670, *R^2^* = 0.818). The regularized linear models performed comparably to each other (*R^2^* ≈ 0.78–0.79) but were inferior to the tree-based ensembles, whereas the single decision tree had the lowest performance (RMSE = 0.813, *R^2^* = 0.732). In repeated 5-fold cross-validation, the random forest achieved a mean RMSE of 0.631, consistent with its test-set RMSE of 0.666. The random forest showed a modest training-to-test gap (training *R^2^* = 0.967 vs. test *R^2^* = 0.820). Based on these evaluation metrics, the optimized Random Forest was selected as the final model, and its back-transformed test-set RMSE was 1,408.6 yuan.

**Table 2:** Comparison of performance indicators for different models of total TCM cost.

Model	RMSE	R^2^	MAE	RMSE (yuan)
Random forest (Optimized)	0.666	0.820	0.353	1408.6
XGBoost	0.670	0.818	0.366	1512.6
Elastic net	0.725	0.787	0.389	1683.6
Lasso regression	0.725	0.787	0.387	1657.7
Linear regression	0.725	0.787	0.395	1756.2
Ridge regression	0.730	0.784	0.407	1848.4
Decision tree	0.813	0.732	0.441	1814.6

RMSE, root mean square error; MAE, mean absolute error; R^2^, coefficient of determination. The outcome was log(1 + cost)-transformed; RMSE, MAE and R^2^ are reported on the log-transformed scale, and RMSE in yuan was obtained after back-transformation of the log-scale predictions. Lower RMSE and MAE and higher R^2^ indicate better performance.

Figure 1 illustrates the predictive performance and interpretability of the random forest model for total TCM cost. Both model-based %IncMSE importance (Figure 1a) and aggregated SHAP importance (Figure 1b) consistently ranked the total cost of TCM treatment items, length of hospital stay and the total cost of Chinese herbal medicines as the three dominant predictors, with little contribution from demographic and clinical factors. The full ranking and numeric values of the permutation importance and SHAP importance are provided in Tables 3, 4, respectively. Importantly, the cumulative importance of these top three features alone contributed approximately 81% of the total SHAP importance, far more than all other variables combined. The SHAP summary plot (Figure 1c) also showed directional effects: larger values of core cost-related features were strongly associated with increased predicted total TCM costs, while other variables showed heterogeneous, non-linear associations. The model demonstrated satisfactory explanatory accuracy with *R^2^* = 0.820 and RMSE = 0.666 on the log scale (back-transformed RMSE = 1,408.6 yuan) (Figure 1d), explaining around 82% of the variance in log-transformed total TCM costs with only minor deviations at the high-cost end. The scatter plot of predicted versus actual values demonstrated a tight clustering of points along the 1:1 line, with residuals evenly distributed across the range of observed costs, indicating no substantial systematic bias and confirming the model’s robust performance across different cost strata.

**Figure 1:**
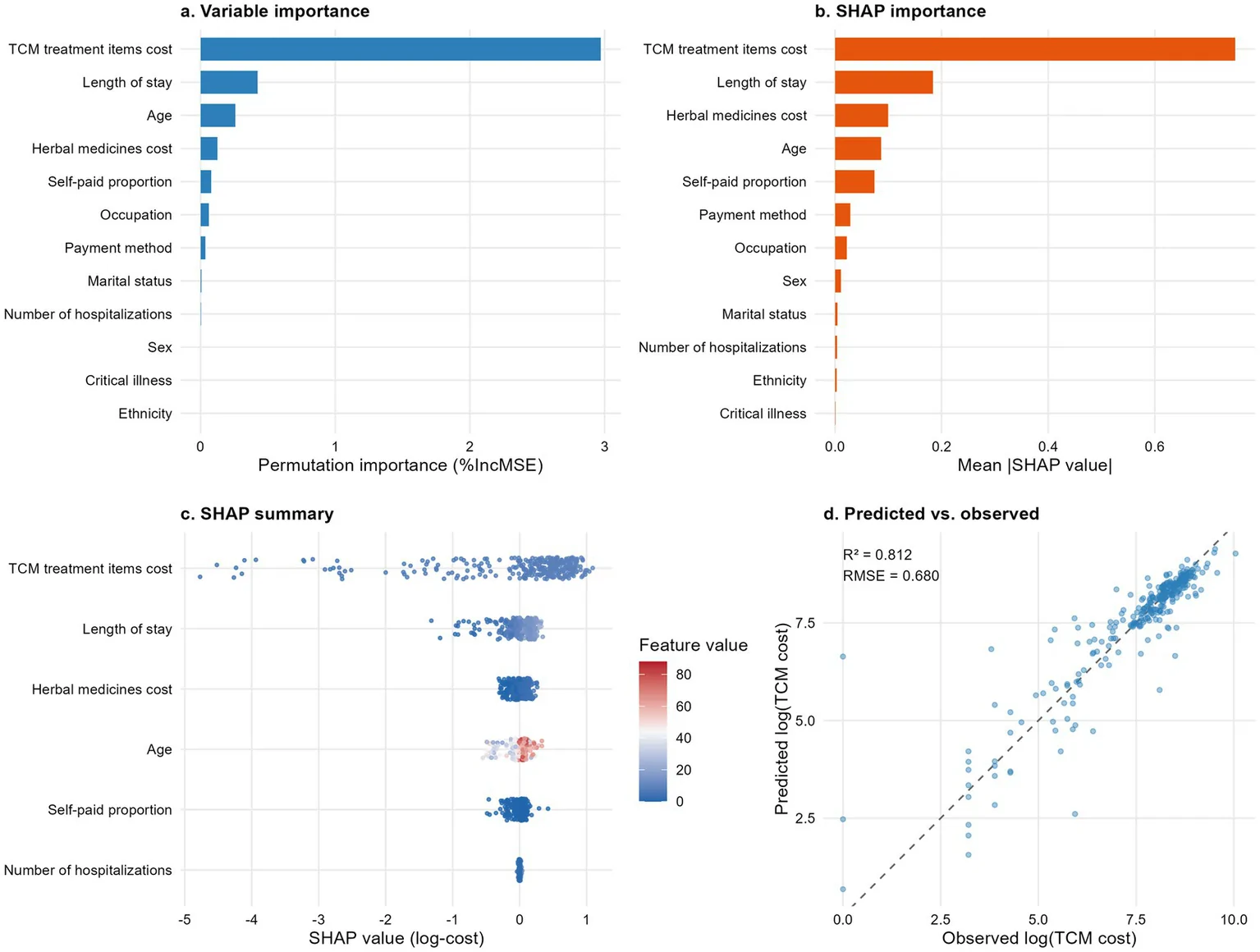
Predictive performance and SHAP-based interpretability of the random forest model for total TCM cost. **(a)** Top-left: Random forest feature importance ranked by the percentage increase in mean squared error (%IncMSE); **(b)** Top-right: Aggregated SHAP feature importance based on the averaged absolute SHAP values across all samples; **(c)** Bottom-left: SHAP summary dot plot presenting the magnitude of feature impacts and the distribution of individual feature values; **(d)** Bottom-right: Scatter plot of model-predicted log-transformed total TCM cost against observed values, with model fitness indices (R^2^ = 0.820) and RMSE = 0.666 annotated.

**Table 3:** Random forest variable importance ranking for the total TCM cost model.

Rank	Variable	%IncMSE
1	Total cost of TCM treatment items	2.971
2	Length of hospital stay	0.424
3	Age	0.259
4	Total cost of Chinese herbal medicines	0.127
5	Proportion of out-of-pocket expenses	0.079
6	Occupation	0.062
7	Payment method	0.035
8	Marital status	0.006
9	Number of hospitalizations	0.003
10	Sex	0.002
11	Critical illness status	0.0002
12	Ethnicity	−0.0003

%IncMSE, percentage increase in mean squared error from permutation of each variable in the optimized random forest model. Higher values indicate greater variable importance; a negative value indicates that permuting the variable slightly improved prediction.

**Table 4:** SHAP variable importance ranking for the total TCM cost model.

Rank	Variable	Mean |SHAP value|
1	Total cost of TCM treatment items	0.7508
2	Length of hospital stay	0.1836
3	Total cost of Chinese herbal medicines	0.0997
4	Age	0.0868
5	Proportion of out-of-pocket expenses	0.0742
6	Payment method	0.0288
7	Occupation	0.0225
8	Sex	0.0112
9	Marital status	0.0049
10	Number of hospitalizations	0.0039
11	Ethnicity	0.0028
12	Critical illness status	0.0010

Mean |SHAP value|, mean absolute Shapley additive explanation value across all samples, computed on the log-transformed scale. Higher values indicate greater contribution to model predictions.

## 4 Discussion

### 4.1 Principal findings

In this retrospective study of 1,532 inpatient medical records from a TCM department, we identified the drivers of total TCM cost through comprehensive model comparison. The optimized Random Forest model outperformed regularized linear models and XGBoost, achieving a test *R^2^* of 0.820 on the log-transformed scale and a back-transformed RMSE of 1,408.6 yuan. Permutation importance and SHAP analysis consistently identified the total cost of TCM treatment items as the dominant predictor, followed by length of hospital stay and the total cost of Chinese herbal medicines, whereas demographic and clinical factors contributed little to the model.

### 4.2 Interpretation of cost-component importance

The total cost of TCM treatment items was by far the most important predictor of total TCM cost. This suggests that non-pharmacological TCM services, such as acupuncture, tuina, moxibustion, acupoint application, and related TCM-specific procedures, represent the principal component of inpatient TCM expenditure in the studied hospital. Because these cost components are part of total TCM cost itself, their high importance reflects the internal cost structure of TCM services rather than an independent causal effect. Nevertheless, this finding is operationally informative: it indicates where variation in TCM expenditure arises and supports more refined cost accounting and standardized management of TCM treatment items, particularly under ongoing medical service price reform and DRG/DIP payment reform (14, 15).

### 4.3 Length of hospital stay and cost variation

Length of hospital stay remained the second most important predictor. This result is consistent with previous evidence showing that hospitalization duration is a major correlate of inpatient costs (16). Longer stays may increase cumulative costs through bed use, nursing care, re-treatments, medication use and daily service charges. However, length of stay should not be interpreted solely as a modifiable causal factor. It may also be indicative of disease severity, treatment complexity, delayed recovery or discharge planning constraints. Hence, cost-control strategies should aim at reducing unnecessary hospitalization while ensuring clinical safety and continuity of care (17).

### 4.4 Payment method, out-of-pocket burden and patient characteristics

Payment method and out-of-pocket burden made small but non-negligible predictive contributions to total TCM cost, which may reflect differences in reimbursement rules, patient affordability and medical service utilization. However, the detailed reimbursement ratios, benefit packages, patient income or socioeconomic status were not included in this study. Therefore, the relationships observed should be interpreted as predictive relationships rather than causal evidence. Future research should include more detailed insurance and socioeconomic variables to better evaluate the financial burden of TCM inpatient services (18, 19).

Descriptive analyses showed differences in total TCM cost by sex, marital status, ethnicity, occupation and critical illness status. These differences may be due to unmeasured socioeconomic, geographic, cultural, health-seeking and healthcare access factors. Due to the single-center design and limited clinical covariates, these findings should be regarded as exploratory. Further multicenter studies with detailed socioeconomic and clinical data are needed to clarify whether these characteristics independently contribute to cost variation (20, 21).

### 4.5 Model performance, skewness handling and overfitting

Consistent with the right-skewed distribution of medical costs, the outcome and the skewed cost-component predictors were log(1 + x)-transformed, and model performance was reported on both the log-transformed and the back-transformed yuan scales. The tree-based ensembles (random forest and XGBoost) outperformed the regularized linear models, consistent with their ability to capture the nonlinear and heterogeneous structure of cost data. To address overfitting concerns for tree models, we used repeated 5-fold cross-validation (5 repeats) for hyperparameter tuning and performance estimation, retained an independent hold-out test set, and reported training, cross-validation and test performance separately. The random forest showed a modest training-to-test gap (training *R^2^* = 0.967 vs. test *R^2^* = 0.820), and its cross-validation RMSE (0.631) was consistent with its test-set RMSE (0.666), indicating that the test-set estimate was stable.

### 4.6 Interpretation of SHAP results

SHAP values quantify values quantify each variable’s contribution to the model’s predictions and therefore reflect associations learned by the model, not causal relationships. Throughout this study, SHAP results are interpreted as predictive importance and directional associations rather than as evidence of causal effects. This is particularly important because the two leading predictors are themselves cost components, so their high SHAP importance partly reflects the arithmetic relationship between total TCM cost and its components.

### 4.7 Implications for TCM service management

The findings have several practical implications. First, TCM treatment items should be included as key components in cost accounting and internal cost monitoring systems for TCM departments. Second, hospitals should strengthen standardized management of TCM-specific services to reduce unwarranted variation in service use and cost. Third, under DRG/DIP payment reform, payment and pricing policies should consider the distinct cost structure of TCM services and avoid applying a uniform cost-control logic without considering service characteristics. Finally, hospital administrators may use real-world data-based analytical tools as supplementary instruments for cost monitoring and the identification of high-cost patterns, provided that the models are externally validated and appropriately interpreted.

### 4.8 Strengths and limitations

This study has multiple strengths. Based on real-world inpatient records from a TCM department, we constructed an optimized Random Forest model to explain total TCM cost. The analysis followed a reproducible workflow including log(1 + x) transformation of a skewed outcome, comparison of seven algorithms, repeated cross-validation for tuning and performance estimation, an independent hold-out test set, and formal overfitting assessment. Combined with SHAP interpretation, we clarified the contribution and action patterns of core influencing factors, offering empirical evidence for analyzing the cost composition of inpatient TCM services in tertiary hospitals.

Several limitations exist in this work. First, it is a single-center retrospective study, which restricts the generalizability of the conclusions. Second, the model incorporates direct cost-component indicators as predictors, so it is applicable for interpreting cost structure rather than prospective prediction at admission. Third, major clinical indicators including diagnosis, disease severity, comorbidities and TCM syndrome differentiation were not included in the analysis. Fourth, only internal validation was conducted, and the Random Forest model for TCM cost faced potential overfitting risks due to skewed cost distribution and heterogeneous service utilization. Fifth, feature importance derived from machine learning models only represents predictive relevance, not causal associations. Lastly, we did not exclude repeated admissions of the same patient, which may impair the independence of statistical samples. As an external validation plan, we intend to evaluate the model in independent multicenter cohorts and, where feasible, in a temporally distinct validation set before any clinical or administrative use.

## 5 Conclusion

In this study, we established an optimized Random Forest model to explain total TCM costs of inpatients in a tertiary hospital in China. The total cost of TCM treatment items dominated predictions of total TCM cost, followed by length of hospital stay and the total cost of Chinese herbal medicines. These results demonstrate the substantial contribution of TCM-related services to inpatient expenditure, suggesting the necessity of refined cost accounting and standardized management for TCM treatments. Given the inclusion of cost components as predictors and the lack of external validation, future research should construct admission-based models using baseline clinical variables and conduct multicenter validation to facilitate practical application.

## Data Availability

The original contributions presented in the study are included in the article/Supplementary material, further inquiries can be directed to the corresponding authors.
