# Supplementary material for: Interpretable machine learning for identifying cost drivers of inpatient traditional Chinese medicine services in a tertiary hospital in China
Source: Front Public Health. 2026 Oct 21;14:1937712. doi: 10.3389/fpubh.2026.1937712 (PMC13730953; doi:10.3389/fpubh.2026.1937712)
Supplement: Supplementary file 1 [file Supplementary_file_1.DOCX]

**Supplementary Table Legends**

**Supplementary Table 1.** Variable importance results for the random forest model predicting total TCM cost (permutation importance and SHAP importance).

**Supplementary Table 1. Variable importance results for the random forest model predicting total TCM cost**

| Rank | Variable | %IncMSE | Mean \|SHAP value\| |
| --- | --- | --- | --- |
| 1 | Total cost of TCM treatment items | 2.971 | 0.7508 |
| 2 | Length of hospital stay | 0.424 | 0.1836 |
| 3 | Age | 0.259 | 0.0868 |
| 4 | Total cost of Chinese herbal medicines | 0.127 | 0.0997 |
| 5 | Proportion of out-of-pocket expenses | 0.079 | 0.0742 |
| 6 | Occupation | 0.062 | 0.0225 |
| 7 | Payment method | 0.035 | 0.0288 |
| 8 | Marital status | 0.006 | 0.0049 |
| 9 | Number of hospitalizations | 0.003 | 0.0039 |
| 10 | Sex | 0.002 | 0.0112 |
| 11 | Critical illness status | 0.0002 | 0.0010 |
| 12 | Ethnicity | −0.0003 | 0.0028 |

**Note: %IncMSE, percentage increase in mean squared error from permutation of each variable; Mean |SHAP value|, mean absolute Shapley additive explanation value across all samples (computed on the log-transformed scale). Variables are listed in order of the permutation importance ranking; a negative %IncMSE indicates that permuting the variable slightly improved prediction.**

**Supplementary Figure Legends**

**Supplementary Figure S1.** Flowchart of inclusion and exclusion for study subjects.

**Supplementary Figure S2.** SHAP heatmap of the total TCM cost prediction model.

**Supplementary Figure S3.** Residual and Q-Q plots for the total TCM cost prediction model.

**Supplementary Figure S4.** SHAP dependence plots for the cost of TCM treatment items, length of hospital stay and the cost of Chinese herbal medicines in the total TCM cost prediction model.

**Supplementary Figure S5.** Performance comparison of models for total TCM cost prediction.

**Supplementary Figure S6.** Combined error analysis plots for the total TCM cost prediction model.

**Supplementary Figure S7.** Combined plots for predictive performance of the total TCM cost model.

HIS data of inpatients admitted to the Department of Traditional Chinese Medicine (TCM) of a tertiary hospital in Guiyang from 2023 to 2025 were included（N=1583）

Finally, medical record information of 1,532 hospitalized patients was included.（n=1532）

Outliers of total hospitalization costs were excluded using the IQR method;Exclude cases with the proportion of out-of-pocket expenses outside 0–1.(n=1570)

Exclude outliers in statistics and clinical data, and cases with missing core information.(n=1532)

# **Supplementary Figure S1.Flowchart of inclusion and exclusion for study subjects.**

**Supplementary Figure S2.SHAP Heatmap of the Total TCM Cost Prediction Model.**

**Supplementary Figure S3.Residual and Q-Q Plots for the Total TCM Cost Prediction Model.**

**Supplementary Figure S4.SHAP Dependence Plots for the Cost of TCM Treatment Items, Length of Hospital Stay and the Cost of Chinese Herbal Medicines in the Total TCM Cost Prediction Model.**

**Supplementary Figure S5.Performance Comparison of Models for Total TCM Cost Prediction.**

**Supplementary Figure S6.Combined Error Analysis Plots for the Total TCM Cost Prediction Model.**

**Supplementary Figure S7.Combined Plots for Predictive Performance of the Total TCM Cost Model.**

# **Supplementary Figure S8.Combined Plots for Predictive Performance of the Total TCM Cost Model**
